# Several Metabolite Families Display Inflexibility during Glucose Challenge in Patients with Type 2 Diabetes: An Untargeted Metabolomics Study

**DOI:** 10.3390/metabo13010131

**Published:** 2023-01-15

**Authors:** Giovanni Fanni, Jan W. Eriksson, Maria J. Pereira

**Affiliations:** Department of Medical Sciences, Clinical Diabetes and Metabolism, Uppsala University, 751 85 Uppsala, Sweden

**Keywords:** type 2 diabetes, oral glucose tolerance test, metabolomics

## Abstract

Metabolic inflexibility is a hallmark of insulin resistance and can be extensively explored with high-throughput metabolomics techniques. However, the dynamic regulation of the metabolome during an oral glucose tolerance test (OGTT) in subjects with type 2 diabetes (T2D) is largely unknown. We aimed to identify alterations in metabolite responses to OGTT in subjects with T2D using untargeted metabolomics of both plasma and subcutaneous adipose tissue (SAT) samples. Twenty subjects with T2D and twenty healthy controls matched for sex, age, and body mass index (BMI) were profiled with untargeted metabolomics both in plasma (755 metabolites) and in the SAT (588) during an OGTT. We assessed metabolite concentration changes 90 min after the glucose load, and those responses were compared between patients with T2D and controls. Post-hoc analyses were performed to explore the associations between glucose-induced metabolite responses and markers of obesity and glucose metabolism, sex, and age. During the OGTT, T2D subjects had an impaired reduction in plasma levels of several metabolite families, including acylcarnitines, amino acids, acyl ethanolamines, and fatty acid derivates (*p* < 0.05), compared to controls. Additionally, patients with T2D had a greater increase in plasma glucose and fructose levels during the OGTT compared to controls (*p* < 0.05). The plasma concentration change of most metabolites after the glucose load was mainly associated with indices of hyperglycemia rather than insulin resistance, insulin secretion, or BMI. In multiple linear regression analyses, hyperglycemia indices (glucose area under the curve (AUC) during OGTT and glycosylated hemoglobin (HbA1c)) were the strongest predictors of plasma metabolite changes during the OGTT. No differences were found in the adipose tissue metabolome in response to the glucose challenge between T2D and controls. Using a metabolomics approach, we show that T2D patients display attenuated responses in several circulating metabolite families during an OGTT. Besides the well-known increase in monosaccharides, the glucose-induced lowering of amino acids, acylcarnitines, and fatty acid derivatives was attenuated in T2D subjects compared to controls. These data support the hypothesis of inflexibility in several metabolic pathways, which may contribute to dysregulated substrate partitioning and turnover in T2D. These findings are not directly associated with changes in adipose tissue metabolism; therefore, other tissues, such as muscle and liver, are probably of greater importance.

## 1. Introduction

Type 2 diabetes (T2D) is a complex disease characterized by disturbances affecting the whole metabolism and resulting in diffuse alteration of glucose and lipid homeostasis [1].

Metabolic inflexibility is the inability to adapt to changes in metabolic demand according to prevailing conditions or activity and is a key hallmark of insulin resistance [2]. One of its main pathological implications is that insulin-sensitive tissues cannot shift from catabolic fatty acid oxidative processes during fasting to anabolic glucose oxidative processes in the fed state [2]. Adipose tissue has been recognized as an important player in this pathological process [3]. Metabolic inflexibility is traditionally investigated through indirect calorimetry during hyperinsulinemic clamp [4] and has been associated with cardiometabolic diseases [5].

High-throughput metabolomics techniques allow a comprehensive overview of the metabolic status both in physiologic and pathologic conditions and have revealed several metabolites and pathways that are dysregulated long before the clinical onset of T2D [6,7]. This can help to identify new diagnostic tools for early detection of the disease and new therapeutic targets [8,9].

In this context, many studies have depicted the metabolomics signature of T2D during fasting [10]. Branched-chain amino acid (BCAAs) catabolism is impaired in insulin-resistant subjects, resulting in higher circulating levels of these metabolites [11,12], which interferes with early-stage insulin signaling in insulin-target tissues [13]. Other metabolic pathways are also disrupted in insulin resistance states, such as acylcarnitines and other lipidic molecules [6,9], depicting a complex metabolomics signature for this condition. However, investigating the fasting-state metabolomic signature does not provide information on dynamic changes occurring in the fasted-to-fed transition. Therefore, it is of interest to outline the dynamic metabolomic changes associated with insulin resistance and T2D also in non-fasting conditions. Several studies have performed metabolomics during an oral glucose tolerance test (OGTT) in healthy subjects, showing that a glucose load: (1) decreases circulating levels of FFAs and acylcarnitines, reflecting a switch from beta-oxidation to glycolysis and lipid storage; (2) increases circulating levels of bile acids and lysophosphatidylcholines as markers of induced enterohepatic circulation of bile acids and redistribution of phospholipids [14,15,16]. To the best of our knowledge, only one study has directly compared the changes in the untargeted plasma metabolome upon a glucose load between subjects with and without T2D [17], but none has been carried out with a parallel comparison on the metabolome of the subcutaneous adipose tissue (SAT). This study aimed to identify metabolic alterations during an oral glucose load in subjects with T2D using an untargeted metabolomics and lipidomics approach on both plasma and SAT samples. This setup provides a feasible method of describing metabolic inflexibility using the metabolomic technique.

## 2. Methods

### 2.1. Subjects and Study Design

Briefly, 20 subjects with T2D (10 males and 10 females) and 20 controls matched for sex, age (58 ± 9 vs. 58 ± 11 years), and BMI (30.7 ± 4.9 vs. 30.8 ± 4.6 kg/m^2^) were recruited. T2D was diagnosed according to the ADA criteria. All subjects with T2D were on treatment with only metformin for at least 3 months before the study, with doses ranging from 500 to 2500 mg according to clinical guidelines. Further characteristics of this cohort have been previously described [18] and are summarized in Table 1. The study was approved by the Regional Ethics Review Board in Uppsala (Dnr 2013/183 and 2013/494), and all participants gave their written informed consent.

The subjects visited the research facility (Uppsala University Hospital) after an overnight fast. First, a medical assessment was performed, and anthropometric data were collected. Secondly, fasting blood samples were collected and analyzed for plasma glucose and lipids, serum insulin, and C-peptide at the Department of Clinical Chemistry, Uppsala University Hospital. Afterwards, a needle biopsy of the SAT was taken in the fasting state from the lower part of the abdomen after local anesthesia with lidocaine (Xylocaine; AstraZeneca, Södertälje, Sweden). Then, an OGTT was performed to analyze plasma glucose and serum insulin via the administration of a bottle of a solution containing 75 g of glucose. Another SAT needle biopsy and a blood sample were taken 90 min after glucose administration with the same abovementioned procedure. Biopsies were taken from different sides of the abdomen to avoid local tissue injury or inflammation at the biopsy site. Part of the fasting and the post-OGTT SAT biopsy samples were snap-frozen in liquid nitrogen and used for metabolomics [18].

### 2.2. Metabolites Quantification

Metabolite and lipid quantification was performed by Metabolon, Inc.’s (Durham, NC, USA) TrueVision™ analysis, which includes the global mVision platform and TrueMass^®^ Lipomic Panel. The amounts provided for analysis were 2 × 150 µL of serum plus 1 × 100 mg and 1 × 50 mg of adipose tissue (whole-tissue biopsy) per sample for Metabolon’s mVision and TrueMass Lipomics analysis, respectively. Plasma and adipose tissue samples were analyzed at fasting and 90 minutes after glucose ingestion. All samples were processed by Metabolon (Research Triangle Park, NC, USA) using GC/MS and LC/MS/MS platforms with the methodological details previously reported [18] and summarized as follows:

**Sample Preparation.** Samples were prepared using the automated MicroLab STAR system from Hamilton Co (Reno, NV, USA). Recovery standards were added prior to the first step in the extraction process for quality control (QC) purposes. Sample preparation was conducted using a methanol extraction to remove the protein fraction while allowing maximum recovery of small molecules. The resulting extract was divided into five fractions: one for analysis by UPLC-MS/MS with positive ion mode electrospray ionization, one for analysis by UPLC-MS/MS with negative ion mode electrospray ionization, one for liquid chromatography (LC) polar platform, one for analysis by gas chromatography/mass spectrometry (GC/MS), and one as a backup. Samples were placed briefly on a TurboVap (Zymark) under nitrogen to remove the organic solvent. For LC, the samples were stored under nitrogen overnight. For GC, the samples were dried under a vacuum overnight. Samples were then prepared for the appropriate instrument, either LC/MS or GC/MS.

**Liquid Chromatography/Mass Spectrometry (LC/MS, LC/MS2) Analysis.** The sample extract for LC/MS/MS was split into two aliquots, dried, and then reconstituted in acidic or basic LC-compatible solvents, each containing eight or more injection standards at fixed concentrations. One aliquot was analyzed using acidic positive ion optimized conditions, and the other using basic negative ion optimized conditions in two independent injections using separate dedicated columns. The MS analysis alternated between MS and data-dependent MS2 scans using dynamic exclusion.

**Gas Chromatography/Mass Spectrometry (GC/MS) Analysis.** GC/MS sample extracts were re-dried under vacuum desiccation for a minimum of 18 h prior to being derivatized under dried nitrogen using bistrimethyl-silyl-triflouroacetamide (BSTFA). The GC column was 5% phenyl, and the temperature ramp was from 60 °C to 340 °C in 17.5 min. Samples were analyzed on a Thermo-Finnigan Trace DSQ fast-scanning single quadrupole mass spectrometer using electron impact ionization and operated at unit mass resolving power. The instrument was tuned and calibrated for mass resolution and mass accuracy on a daily basis.

**Accurate Mass Determination (LC/MS) and MS/MS Fragmentation (LC/MS/MS) for Structural Elucidation**. The LC/MS portion of the platform was based on a Wates ACQUITY UPLC and a Thermo-Finnigan OrbiElite mass spectrometer, which had a linear ion-trap (LIT) front end and an orbitrap mass spectrometer backend. Accurate mass measurements could be made on the parent ion as well as fragments. The typical mass error was less than 5 ppm. Fragmentation spectra (MS^n^) were targeted.

**Metabolite Identification.** Compounds were identified by comparison to library entries of purified standards or recurrent unknown entities. Identification of known chemical entities was based on comparison to metabolomics library entries of more than 3300 commercially available purified standards, and additional presently unknown entities were identified by their recurrent nature.

**Metabolite Quantification and Data Normalization.** Peaks were quantified using the area under the curve. For studies spanning multiple days, a data normalization step was performed to correct variation resulting from instrument inter-day tuning differences. Essentially, each compound was corrected in run-day blocks by registering the medians to equal one (1.00) and normalizing each data point proportionately. Missing values were assumed to result from areas falling below the detection limits. As such, missing values were imputed with the observed minimum for each metabolite after the normalization step.

**TrueMass Lipomics Panel**. Lipids were extracted in the presence of authentic internal standards by the method of Folch [19] using chloroform: methanol (2:1 *v*/*v*). For separating neutral lipid classes (free fatty acids; triglycerides; diglyceride; cholesteryl ester) a solvent system consisting of petroleum ether/diethyl ether/acetic acid (80:20:1) was employed. Individual phospholipid classes within each extract (phosphatidylcholine; phosphatidylethanolamine) were separated using the Agilent Technologies 1100 Series LC (Agilent Technologies, Santa Clara, CA, USA). Each lipid class was transesterified in 1% sulfuric acid in methanol in a sealed vial under a nitrogen atmosphere at 100 °C for 45 min. The resulting fatty acid methyl esters were extracted from the mixture with hexane containing 0.05% butylated hydroxytoluene and prepared for GC by sealing the hexane extracts under nitrogen. Fatty acid methyl esters were separated and quantified by capillary GC (Agilent Technologies 6890 Series GC) equipped with a 30 m DB 88 capillary column (Agilent Technologies) and a flame detector.

Metabolite levels were quantified in arbitrary units (AU), while fatty acid derivatives were quantified in mole percentage of the lipidic subfamily. Metabolites missing in more than 20% of the samples among control and T2D groups were excluded from the data (modified 80% rule) [19]. The remaining missing metabolites that failed to reach the detection threshold were imputed from the minimum observed value of the metabolite.

### 2.3. Pathway Analysis and Statistical Analysis

All data are presented as mean ± SEM unless otherwise indicated. Statistical analyses were performed using Metaboanalyst 5.0 (http://www.metaboanalyst.ca, accessed on 15 November 2022) and Prism 9 (GraphPad, San Diego, CA, USA).

We calculated the ratio between the post-OGTT and the fasting level of all metabolites with absolute quantification in arbitrary units (fold-change). We calculated the difference between the post-OGTT and the fasting percentage of all lipids with mole percentage quantification. Multiple Mann–Whitney tests were used to compare fasting metabolite levels and the changes after the glucose load versus fasting levels in all metabolites between subjects with and without T2D. *p*-values were adjusted with the Benjamini, Krieger, and Yekutieli false discovery rate procedure to account for multiple testing. Significantly different metabolites were then detected based on a *Q*-value threshold <0.05. Spearman’s correlations were performed to test the association between OGTT-induced fold changes and several clinical and metabolic variables. Principal component analysis (PCA) was used to condense all metabolite profiles into one principal component (PC1) that explained 47% of the observed variance. The PC1 scores were used as outcomes in multiple regression models to identify the clinical variables associated with the changes in metabolite levels after OGTT. *p*-values and *Q*-values lower than 0.05 were considered significant.

Metaboanalyst 5.0 was used for automated pathway analysis and interpretation of metabolomics data.

This was an exploratory study with no formal power analyses. However, according to previous studies using a similar approach [15,20], this study has at least 80% power to detect 20% differences in the effects of OGTT on metabolite concentrations between control and T2D groups. Still, no adjustment for multiplicity was considered for this purpose.

## 3. Results

### 3.1. Untargeted Metabolomics and Lipidomics during the OGTT in Controls and Type 2 Diabetes Subjects

We identified 541 metabolites with absolute quantification and 214 lipids with mole percentage quantification in plasma. In the adipose tissue, we found 365 metabolites with absolute quantification and 223 lipids with mole percentage quantification. We did not consider those molecules that could not be identified as known metabolites. The complete metabolite list with their mean fold change 90 min after glucose administration is available in Supplementary Materials File S1.

**Figure 1:**
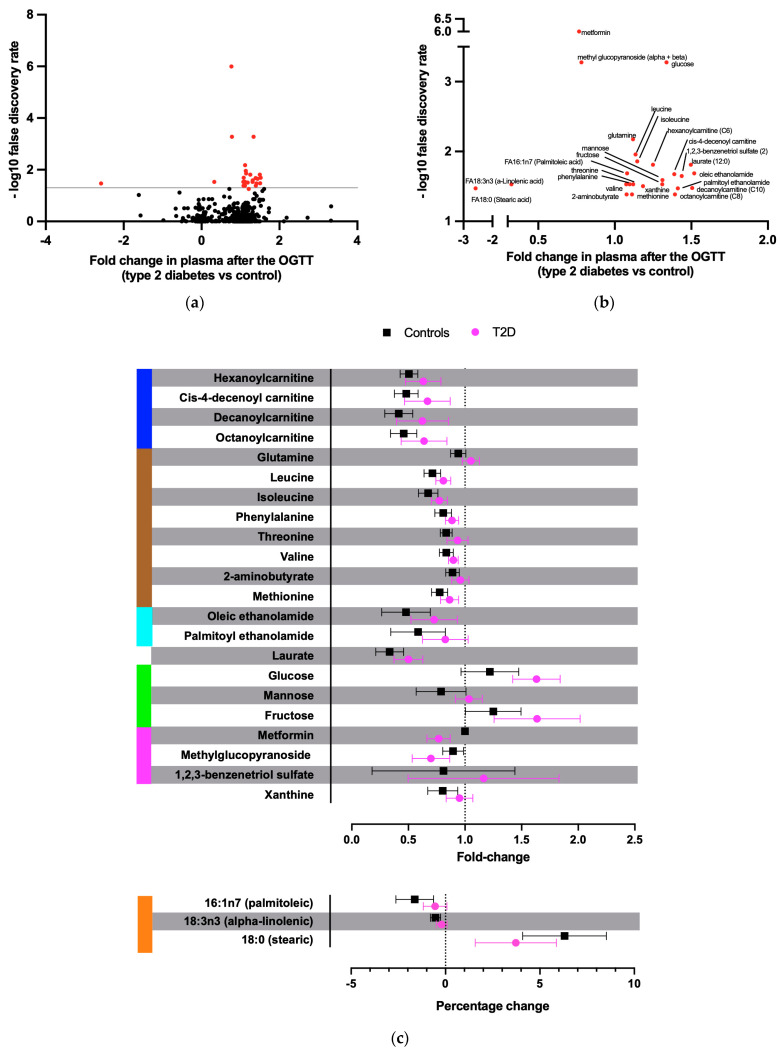
Metabolites with a significantly different fold change after the OGTT between T2D and control subjects. (**a**) Volcano plot of the differences in plasma metabolite concentration changes 90 min after 75 g glucose ingestion between patients with T2D and controls. Y-axis shows –log_10_ false discovery rate. Red dots depict significant results. (**b**) Zoom-out on the significant metabolites from panel (**a**). (**c**) Fold and percentage changes of the significant metabolites after the OGTT for T2D and control subjects. In blue, acylcarnitines; in brown, amino acids; in light blue, acyl ethanolamides; in green, monosaccharides; in magenta, xenobiotics; in orange, free fatty acids.

**Figure 2:**
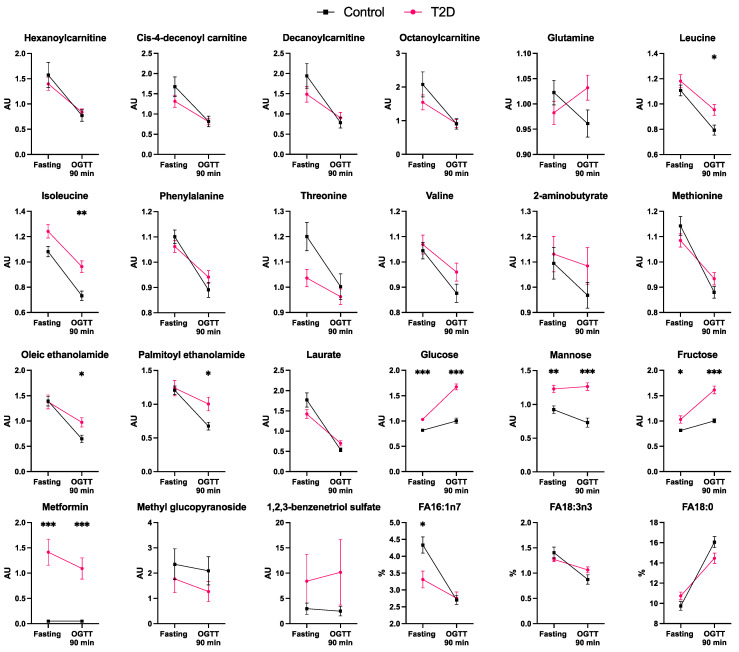
Fasting and 90 min post-OGTT plasma levels of the metabolites that significantly differ in response to the OGTT between control and T2D subjects (*n* = 20 per each group). FA16:1n7: palmitoleic acid. FA18:0: stearic acid. FA18:3n3: alpha-linoleic acid. Data are presented as mean ± SEM. AU, arbitrary units. %, percentage of the free fatty acid family total concentration. * *p* < 0.05, ** *p* < 0.01, *** *p* < 0.001 for differences between controls and T2D individuals (Mann–Whitney tests).

Levels of 25 unique plasma metabolites were differently affected by the OGTT in T2D compared to controls (*p* < 0.05, after false discovery rate (FDR) correction, Figure 1a,b. In controls, the glucose load caused a reduction in the plasma concentration of several acylcarnitines, acyl ethanolamines, xanthine, mannose, BCAAs, and other amino acids, but this effect was significantly blunted in patients with T2D (all *p* < 0.05 after FDR correction; Figure 1c and Figure 2). Additionally, patients with T2D had a greater rise in plasma glucose and fructose levels during the OGTT compared to controls (both *p* < 0.05). Patients with T2D had a reduced relative reduction of the unsaturated palmitoleic and alpha-linolenic acids and a reduced relative increase of the saturated stearic acid compared to controls (Figure 1c and Figure 2).

Glucose, mannose, and metformin had significantly different plasma concentrations at fasting between T2D and controls after FDR correction (Supplementary Materials File S2).

The alterations observed in adipose tissue and plasma differed largely. In adipose tissue, several metabolites tended to increase during the OGTT in T2D subjects compared to controls, such as lipids, glutamate, and mannose (Supplementary Materials File S2), but did not cross the threshold of statistical significance after FDR correction.

### 3.2. Metabolic Pathways Associated with Abnormal Response to Oral Glucose in Type 2 Diabetes

**Figure 3:**
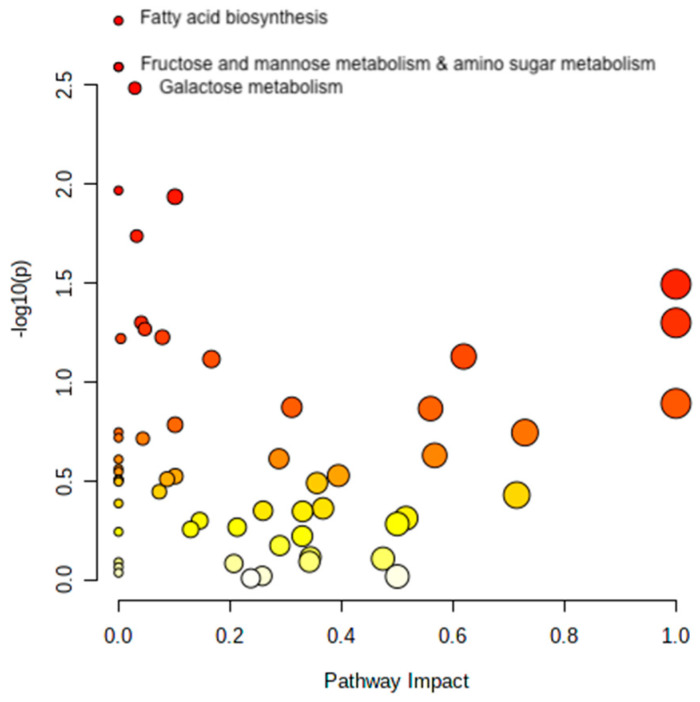
Pathway analysis. The figure shows the pathways that significantly differ in response to the OGTT between controls and T2D subjects. Metabolites are arranged according to the scores based on enrichment analysis (*y*-axis, *Q*-values) and topology analysis (*x*-axis, arbitrary unit). The color of each circle shades from white to red according to the *p*-value, and the size is based on the pathway impact values. Only significant pathways are named in the figure. Figure created with Metaboanalyst 5.0.

The metabolites with different plasma concentration changes during the OGTT in T2D and control subjects were analyzed with automated pathway analyses. The majority of metabolites that differed between controls and T2D were related to fatty acid biosynthesis; fructose, mannose, and galactose metabolism; amino sugar and amino nucleotide metabolism (all *Q* < 0.049) (Figure 3).

### 3.3. Associations with Clinical Parameters

**Figure 4:**
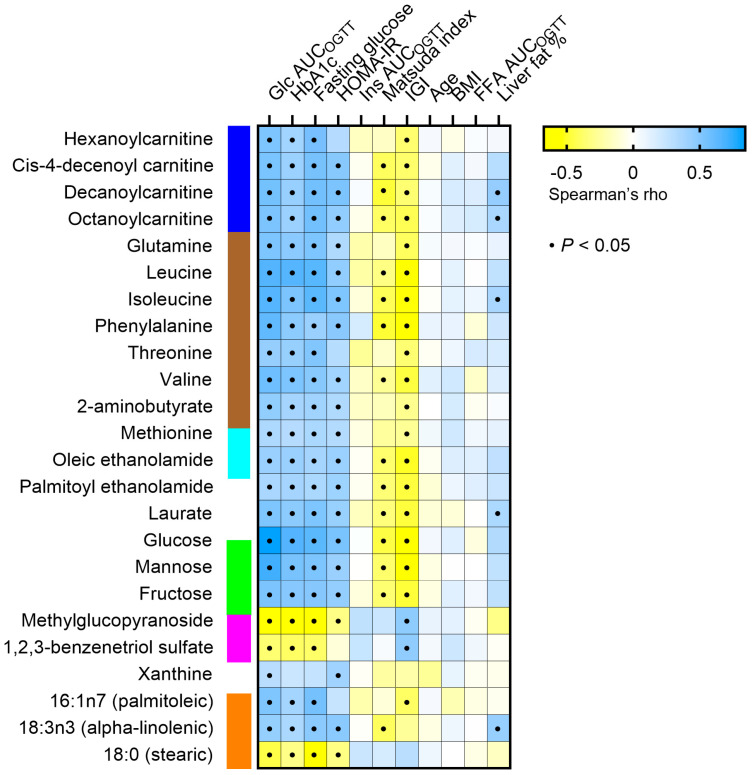
Correlation matrix between plasma concentration change of metabolites that significantly differ in response to the OGTT between controls and T2D individuals (rows) and several clinical parameters (columns). A black dot indicates a statistically significant correlation between the respective metabolite and parameter (*p* < 0.05). Glc AUC_OGTT_: glucose area under the curve during the OGTT. HbA1c, glycosylated hemoglobin. Ins AUC_OGTT_: insulin area under the curve during the OGTT. IGI: insulinogenic index. FFA: free fatty acid. In blue, acylcarnitines; in brown, amino acids; in light blue, acyl ethanolamides; in green, monosaccharides; in magenta, xenobiotics; in orange, free fatty acids.

To screen which clinical parameters were most associated with the metabolites changes during the oral glucose load, we performed a correlation matrix including markers of hyperglycemia, insulin resistance, and insulin production (glucose AUC_OGTT_, HbA1c, fasting glucose, HOMA-IR, insulin AUC_OGTT_, Matsuda index, Insulinogenic index), obesity markers (BMI, FFA AUC_OGTT_, liver fat percentage), and age (Figure 4). Changes in the levels of acylcarnitines, amino acids, and monosaccharides were prominently positively correlated with markers of hyperglycemia, i.e., glucose AUC_OGTT_, HbA1c, and fasting glucose, and the insulin resistance marker HOMA-IR and negatively correlated with IGI. Some of the metabolites were also negatively associated with the Matsuda index. Among the amino acids, BCAAs showed the strongest associations. No remarkable correlations were found between metabolite concentration changes and insulin AUC_OGTT_, age, BMI, FFA AUC_OGTT_, or liver fat content.

In order to identify whether hyperglycemia, insulin resistance, or obesity predicted the observed changes in plasma metabolites, relevant parameters were included in multivariate regression analyses (Table 2). PCA was used to condense all metabolites into one principal component, and the PC scores were used as the dependent variable (Table 2). The models were also adjusted for sex and age. Only glucose AUC_OGTT_ or HbA1c were significant predictors of the PC scores derived from the metabolites (model 1 r^2^ = 0.61, *p* < 0.001 and model 2 r^2^ = 0.47, *p* < 0.001, respectively) (Table 2), while HOMA-IR, BMI, age, and sex were not.

## 4. Discussion

Our results suggest that after an OGTT, T2D subjects display an impaired reduction in plasma levels of several metabolite families, including amino acids, acylcarnitines, and fatty acids, while glucose and fructose levels are increased. Furthermore, we demonstrated that the OGTT-induced metabolome changes are independently associated with hyperglycemia per se rather than insulin resistance.

Evidence about the impact of hyperglycemia and insulin resistance on metabolome changes in the fasted-to-fed transition is poor and fragmented [14,15,21,22]. In the present study, we characterized the global metabolome at fasting and 90 min after a 75 g load of oral glucose, and we observed whether the fold change of the concentration of each metabolite from fasting was different between subjects with and without T2D, with a case-control approach. In response to the glucose challenge, most lipids, acylcarnitines, amino acids, fatty acids, and fatty acid oxidation intermediates decreased in plasma and adipose tissue in control and T2D subjects. As observed in previous studies, these alterations reflect the switch from fatty acid oxidation to glucose oxidation and fat storage during the OGTT [14]. However, after FDR correction, we identified 25 metabolites that showed different dynamics between T2D subjects and controls over a glucose load in plasma but not in adipose tissue. T2D patients display higher plasma monosaccharide levels during an OGTT but attenuated responses in several metabolite families, including amino acids, acylcarnitines, and fatty acid derivatives. The post-OGTT fold changes of amino acids, acylcarnitines, fatty acids, and monosaccharides were positively associated with glucose AUC during the OGTT and HbA1c levels, suggesting that hyperglycemia influences metabolic inflexibility in response to a glucose load. This can also indicate that the metabolic inflexibility during an OGTT in T2D subjects could be due to the reduced amount of glucose entering the tissues [23] and being available for oxidation and storage. This would be consistent with a previous study demonstrating that metabolic flexibility in response to glucose in subjects with T2D is not impaired after controlling for glucose disposal rate [23,24]. Metabolomics research has shown that metformin influences several metabolites’ plasma levels, including reduced concentration of unsaturated lipids, lysophosphatidylcholines, urea cycle metabolites, and purine derivatives [25]. Therefore, even though the patients with T2D did not take metformin on the day of the examination, we cannot rule out a possible impact of metformin in the metabolomics findings. Additionally, treatment with metformin in patients with T2D might have attenuated the investigated differences by lowering the subjects’ insulin resistance in the long term. Although many metabolites are known to be associated with T2D [10], we are the first to report the dynamic changes following an oral glucose load both in plasma and in the SAT. The data presented with this untargeted metabolomics approach suggest that analyses of key metabolites, such as BCAAs, acylcarnitines, monosaccharides, and related pathways, during an OGTT provide a great opportunity to study metabolic inflexibility across different degrees of hyperglycemia and insulin resistance.

**Glucose** and **metformin** levels validated our data. As expected, subjects with T2D had a higher increase in glucose levels after the glucose load than controls [18]. Additionally, since all patients with diabetes were on metformin, a reduced concentration of the drug was appreciable in the second sampling after the glucose load. Patients with T2D did not take metformin the morning before the visit. Still, metformin levels at the time of sampling reflected the pharmacokinetic processes of distribution and metabolism, in line with the known half-time of the drug (2–6 h) [26]. No metformin was detected in the control subjects.

The most numerous metabolite family was amino acids. An impaired reduction in the plasma levels of the BCAAs **leucine, isoleucine, and valine** during the OGTT occurred in T2D subjects. Elevations in BCAAs are considered predictive biomarkers of impaired fasting glucose [11]. BCAAs alter key metabolic processes affecting glucose homeostasis, for instance, activating the mTOR pathway and disrupting insulin signaling [27,28]. The observed impaired reduction of BCAAs during the OGTT in T2D may be due to impaired BCAA catabolism [29] or reduced uptake into the skeletal muscle [27,28,30]. BCAAs excess might spill over into other tissues, including the liver and beta-cells, leading to TCA cycle anaplerosis via BCAAs catabolite products (i.e., alpha-ketoacids), impaired beta-oxidation and higher acylcarnitine release, mitochondrial stress, and disruption of insulin signaling, thus reinforcing hyperglycemia [31,32].

Concomitantly, we found that other amino acids, including **phenylalanine, threonine, and glutamine**, showed an impaired reduction during the glucose challenge in T2D subjects compared to controls.

Impaired changes for BCAAs, phenylalanine, and threonine have been previously reported for obese and insulin-resistant subjects in separate studies [21,22,33,34]. Increased fasting levels of the aromatic amino acid **phenylalanine** and **threonine** have also been associated with diabetes [35,36,37,38,39,40,41]. Recent evidence employing the Mendelian randomization method has confirmed a causal role of several amino acids’ disrupted metabolism in the development of insulin resistance [42]. Additionally, lower **glutamine** levels or glutamine-to-glutamate ratio are associated with a higher risk of diabetes [36,39,40] and a worse cardiometabolic profile [43]. In our study, the OGTT caused a reduction in circulating glutamine in controls, while an increase was seen in T2D subjects. Since insulin resistance results in a reduced ability of insulin to block muscle protein breakdown after a glucose load [16], increased circulating glutamine levels might depend on the higher release of this amino acid into the circulation as a consequence of impaired blockade of protein breakdown. Our results underline that dynamic changes of BCAAs and other amino acids after a glucose load also reflect metabolic inflexibility in T2D subjects with hyperglycemia.

Four acylcarnitines (**hexanoyl carnitine, cis-4-decenoyl carnitine, decanoyl carnitine, and octanoyl carnitine**) showed an impaired reduction in plasma levels after the OGTT in patients with T2D compared to controls. Acylcarnitines are markers of incomplete fatty acid oxidation [44], and their plasma levels are suppressed during an OGTT in healthy men, indicating a metabolic switch from beta-oxidation to glycolysis and liposynthesis [14]. Higher levels of FFA during the OGTT, as previously reported [18], and reduced suppression of acylcarnitines in the T2D group suggest that the oral glucose load did not suppress FFA release or fatty acid oxidation as much as in the control group. These findings are consistent with animal models [45] and with recent evidence that acylcarnitine plasma levels are less suppressed even in subjects with higher insulin resistance but without diabetes [21].

Plasma **mannose** levels are a surrogate for hepatic glycogenolysis, namely the degradation of glycogen into glucose that is released into the circulation in the fasting state. An oral glucose load elicits an insulin response that blocks hepatic glycogenolysis, resulting in lower mannose levels [46], as observed in the control group of this study. However, this was not seen in T2D subjects who displayed unchanged mannose levels during the OGTT. This indicates that hepatic insulin resistance causes a failure in suppressing hepatic glycogenolysis in the fed state, as shown in rat models [47].

Increased plasma **fructose** levels in patients with T2D during the OGTT likely reflect increased endogenous production of this monosaccharide because of the shunt of excess glucose from glycolysis to the polyol pathway. This pathway is insulin-independent and leads to decreased production of endogenous antioxidants and contributes to microvascular diabetic complications affecting tissues that are freely permeable to glucose, such as the retina [48,49]. Additionally, fructose in excess might compete with glucose in other metabolic pathways, leading to disrupted saccharide turnover.

**Laurate** is a medium-chained fatty acid that rises predominantly during fasting due to lipolysis in the adipose tissue mediated by hormone-sensitive lipase. Insulin-sensitive subjects show a major decrease in FFAs circulating levels after an OGTT because of lipolysis suppression induced by insulin on the adipose tissue [50]. Therefore, higher circulating levels of laurate in T2D patients link impaired insulin sensitivity with continuous fatty acid release in the transition from a fasted to a post-prandial state. After a glucose load, the plasma concentration of **stearic acid** (FA18:0) rises while the levels of the unsaturated **palmitoleic** (FA16:1n7) and **alpha-linolenic acid** (FA8:3n3) are reduced [51,52]. These changes are significantly blunted in T2D. This might be caused by altered re-esterification processes and impaired lipid storage in the adipose tissue rather than by adipose insulin resistance per se, as suggested before [18,53]. Moreover, we could not detect lipidome alterations in the adipose tissue after the OGTT.

**Oleic ethanolamide** and **palmitoyl ethanolamide** are endogenous lipid analogues classified as endocannabinoid-like molecules [54]. These lipid amides are endogenous ligands of peroxisome proliferator-activated receptor (PPAR)-alpha, a nuclear receptor activated mainly in the liver to promote fatty acid beta-oxidation, gluconeogenesis, and ketogenesis during energy deprivation [55]. Oleic ethanolamide has gained interest because of its central anorexigenic effect [54]. Their role in insulin resistance is still to be clarified.

Notably, opposite to what Ho et al. found, we saw increased rather than decreased serotonin levels after OGTT, with no difference between controls and patients with T2D, and we did not see significant changes in **TCA cycle intermediates** after glucose load [15,56]. Moreover, we did not reveal any differences in the suppression of **beta-hydroxybutyrate**, a marker of ketogenesis, between T2D and controls, as suggested before [15,16]. This might depend on the different sampling times, different clinical characteristics of the cohorts, or sample sizes.

Surprisingly, we did not see any significant fold changes in the metabolite pool of the adipose tissue after a glucose load. This might depend on the short period between the two measurements (90 min), but it also suggests that tissues other than the adipose, such as the liver or the skeletal muscles, play a major role in determining the early metabolome response observed in plasma following the oral glucose load.

Metabolic inflexibility is associated with hyperglycemia and insulin resistance, but the cause-effect relationship is not known. The observed metabolic inflexible state with raised fatty acids and amino acids in the post-prandial state encompasses a variety of pathways and mechanisms that can further lead to hyperglycemia and insulin resistance. Increased lipids and BCAA in circulation or non-adipose tissues, such as muscle, liver and pancreas, hamper insulin signaling [28,57]. Additionally, an increased supply of fatty acids can lead to defects in fatty acid oxidation and altered mitochondrial energetics [58]. Several studies have also suggested that the rise in circulating BCAAs, derived by a decline in their catabolism in adipose tissue in metabolically compromised individuals [59], can “spill” into catabolic pathways in muscle and liver and reduce the efficiency of the oxidation of fatty acids and glucose, leading to mitochondrial stress, insulin resistance, and further contributing to hyperglycemia [30]. However, this study included subjects where insulin resistance had already developed, and therefore we cannot rule out that insulin resistance itself plays a primary role in the observed metabolic inflexibility.

## 5. Limitations

This study has some limitations. First, the OGTT metabolome sampling was performed 90 min after glucose ingestion. This time might be too short to highlight some later metabolome perturbations, especially in the adipose tissue. Secondly, our cohort consisted of 40 subjects, which might not give sufficient statistical power to detect smaller differences in the metabolome, especially considering the robust correction we performed for multiple testing. Third, our patients with T2D were on metformin treatment, and the potential effect of it on the outlined results is unknown. However, patients did not take metformin on the days of the visit, so the acute effects of the drug on the presented results are expected to be minimal. Additionally, the degree of physical activity was not available and therefore was not included in the models. Finally, this observational study does not allow us to conclude the causality of the associations presented. Further studies are warranted to shed light on the pathophysiology of metabolic inflexibility in insulin resistance.

## 6. Conclusions

We used a metabolomics approach to show that T2D patients display attenuated responses of several metabolites in plasma during an OGTT. This involves several metabolite families, including amino acids, acylcarnitines, and fatty acid derivatives. These perturbations support inflexibility in several metabolic pathways, which can contribute to dysregulated substrate partitioning and turnover in T2D and seem to be associated with chronic hyperglycemia rather than insulin resistance, secretion, or adiposity. These findings are not directly associated with changes in adipose tissue metabolism. Instead, other tissues, such as muscle and liver, may be more important, and the underlying mechanisms and impact in T2D warrant further studies considering the metabolome variation upon different nutrient challenges. 

## Figures and Tables

**Table 1:** Clinical characteristics of study participants.

	Control	T2D
N (F/M)	10/10	10/10
Age (years)	58 (11)	58 (9)
Diabetes duration (years)	N/A	4 (3)
BMI (kg/m^2^)	30.8 (4.6)	30.7 (4.9)
Waist-to-hip ratio	0.96 (0.07)	0.99 (0.05)
Plasma glucose (mmol/L)	6.0 (0.7)	8.2 (1.5) *
Serum insulin (mU/L)	11.5 (5.2)	15.5 (7.0) *
HbA1c (mmol/mol)	37.3 (3.7)	48.8 (8.6) ***
HOMA-IR	3.08 (1.58)	5.27 (2.86) **

Data are presented as mean (SD). * *p* < 0.05; ** *p* < 0.01; *** *p* < 0.001 relative to control (Mann–Whitney U test).

**Table 2:** Multiple linear regression models of principal component score 1 (PC1) derived from metabolites with significantly different fold changes after the OGTT between T2D and control subjects vs. insulin sensitivity and obesity.

	Standardized Beta	*p*-Value	95% CI	R^2^
**Model 1**				
Glu_AUC_	**−0.736**	**<0.001**	[−0.30, −0.13]	
HOMA-IR	−0.010	0.950	[−0.46, 0.43]	
BMI	−0.222	0.098	[−0.36, 0.03]	
				0.61 ***
**Model 2**				
HbA1c	**−0.463**	**0.003**	[−0.30, −0.07]	
HOMA-IR	−0.289	0.068	[−0.85, 0.03]	
BMI	−0.132	0.379	[−0.32, 0.12]	
				0.47 ***

PC1: proportion of variance = 46.5%. Models are adjusted for sex and age. CI, confidence interval for unstandardized coefficient (lower bound, upper bound); Glu_AUC_, glucose area under the curve; BMI, body mass index; HbA1c, glycosylated hemoglobin. Bold values indicate statistical significance. *** *p* < 0.001.

## Data Availability

The datasets generated during and/or analysed during the current study are not publicly available due to a collaboration agreement with a company but are available from the corresponding author on reasonable request.

## Supplementary Materials

The following supporting information can be downloaded at: https://www.mdpi.com/article/10.3390/metabo13010131/s1. Supplementary Materials File S1: list of all metabolites’ concentration in plasma and SAT before and after OGTT. Supplementary Materials File S2: statistical analyses for differences in all metabolites’ plasma concentration after OGTT between T2D and controls.

## References

[1] Fanni G. (2021). Diabetes Is Not (Only) a Disorder of Glucose Metabolism!. Diabet. Med..

[2] Goodpaster B.H., Sparks L.M. (2017). Metabolic Flexibility in Health and Disease. Cell Metab..

[3] Tareen S.H.K., Kutmon M., Adriaens M.E., Mariman E.C.M., de Kok T.M., Arts I.C.W., Evelo C.T. (2018). Exploring the Cellular Network of Metabolic Flexibility in the Adipose Tissue. Genes Nutr..

[4] Rynders C.A., Blanc S., DeJong N., Bessesen D.H., Bergouignan A. (2018). Sedentary Behaviour Is a Key Determinant of Metabolic Inflexibility. J. Physiol..

[5] Yu E.A., Yu T., Jones D.P., Ramirez-Zea M., Stein A.D. (2020). Metabolomic Profiling After a Meal Shows Greater Changes and Lower Metabolic Flexibility in Cardiometabolic Diseases. J. Endocr. Soc..

[6] Ahola-Olli A.V., Mustelin L., Kalimeri M., Kettunen J., Jokelainen J., Auvinen J., Puukka K., Havulinna A.S., Lehtimäki T., Kähönen M. (2019). Circulating Metabolites and the Risk of Type 2 Diabetes: A Prospective Study of 11,896 Young Adults from Four Finnish Cohorts. Diabetologia.

[7] Wittenbecher C., Guasch-Ferré M., Haslam D.E., Dennis C., Li J., Bhupathiraju S.N., Lee C.-H., Qi Q., Liang L., Eliassen A.H. (2022). Changes in Metabolomics Profiles over Ten Years and Subsequent Risk of Developing Type 2 Diabetes: Results from the Nurses’ Health Study. eBioMedicine.

[8] Newgard C.B. (2017). Metabolomics and Metabolic Diseases: Where Do We Stand?. Cell Metab..

[9] Wildberg C., Masuch A., Budde K., Kastenmüller G., Artati A., Rathmann W., Adamski J., Kocher T., Völzke H., Nauck M. (2019). Plasma Metabolomics to Identify and Stratify Patients with Impaired Glucose Tolerance. J. Clin. Endocrinol. Metab..

[10] Guasch-Ferré M., Hruby A., Toledo E., Clish C.B., Martínez-González M.A., Salas-Salvadó J., Hu F.B. (2016). Metabolomics in Prediabetes and Diabetes: A Systematic Review and Meta-Analysis. Diabetes Care.

[11] Menni C., Fauman E., Erte I., Perry J.R.B., Kastenmüller G., Shin S.-Y., Petersen A.-K., Hyde C., Psatha M., Ward K.J. (2013). Biomarkers for Type 2 Diabetes and Impaired Fasting Glucose Using a Nontargeted Metabolomics Approach. Diabetes.

[12] Wang T.J., Larson M.G., Vasan R.S., Cheng S., Rhee E.P., McCabe E., Lewis G.D., Fox C.S., Jacques P.F., Fernandez C. (2011). Metabolite Profiles and the Risk of Developing Diabetes. Nat. Med..

[13] Patti M.E., Brambilla E., Luzi L., Landaker E.J., Kahn C.R. (1998). Bidirectional Modulation of Insulin Action by Amino Acids. J. Clin. Investig..

[14] Zhao X., Peter A., Fritsche J., Elcnerova M., Fritsche A., Häring H.U., Schleicher E.D., Xu G., Lehmann R. (2009). Changes of the Plasma Metabolome during an Oral Glucose Tolerance Test: Is There More than Glucose to Look At?. Am. J. Physiol. Endocrinol. Metab..

[15] Ho J.E., Larson M.G., Vasan R.S., Ghorbani A., Cheng S., Rhee E.P., Florez J.C., Clish C.B., Gerszten R.E., Wang T.J. (2013). Metabolite Profiles During Oral Glucose Challenge. Diabetes.

[16] Shaham O., Wei R., Wang T.J., Ricciardi C., Lewis G.D., Vasan R.S., Carr S.A., Thadhani R., Gerszten R.E., Mootha V.K. (2008). Metabolic Profiling of the Human Response to a Glucose Challenge Reveals Distinct Axes of Insulin Sensitivity. Mol. Syst. Biol..

[17] Huo S., Sun L., Zong G., Shen X., Zheng H., Jin Q., Li H., Yin H., Lin X. (2021). Changes in Plasma Metabolome Profiles Following Oral Glucose Challenge among Adult Chinese. Nutrients.

[18] Pereira M.J., Skrtic S., Katsogiannos P., Abrahamsson N., Sidibeh C.O., Dahgam S., Månsson M., Risérus U., Kullberg J., Eriksson J.W. (2016). Impaired Adipose Tissue Lipid Storage, but Not Altered Lipolysis, Contributes to Elevated Levels of NEFA in Type 2 Diabetes. Degree of Hyperglycemia and Adiposity Are Important Factors. Metabolism.

[19] Wei R., Wang J., Su M., Jia E., Chen S., Chen T., Ni Y. (2018). Missing Value Imputation Approach for Mass Spectrometry-Based Metabolomics Data. Sci. Rep..

[20] Diamanti K., Cavalli M., Pan G., Pereira M.J., Kumar C., Skrtic S., Grabherr M., Risérus U., Eriksson J.W., Komorowski J. (2019). Intra- and Inter-Individual Metabolic Profiling Highlights Carnitine and Lysophosphatidylcholine Pathways as Key Molecular Defects in Type 2 Diabetes. Sci. Rep..

[21] Nowak C., Hetty S., Salihovic S., Castillejo-Lopez C., Ganna A., Cook N.L., Broeckling C.D., Prenni J.E., Shen X., Giedraitis V. (2018). Glucose Challenge Metabolomics Implicates Medium-Chain Acylcarnitines in Insulin Resistance. Sci. Rep..

[22] Liu L., Feng R., Guo F., Li Y., Jiao J., Sun C. (2015). Targeted Metabolomic Analysis Reveals the Association between the Postprandial Change in Palmitic Acid, Branched-Chain Amino Acids and Insulin Resistance in Young Obese Subjects. Diabetes Res. Clin. Pract..

[23] Eriksson J.W., Visvanathar R., Kullberg J., Strand R., Skrtic S., Ekström S., Lubberink M., Lundqvist M.H., Katsogiannos P., Pereira M.J. (2021). Tissue-Specific Glucose Partitioning and Fat Content in Prediabetes and Type 2 Diabetes: Whole-Body PET/MRI during Hyperinsulinemia. Eur. J. Endocrinol..

[24] Galgani J.E., Heilbronn L.K., Azuma K., Kelley D.E., Albu J.B., Pi-Sunyer X., Smith S.R., Ravussin E. (2008). Metabolic Flexibility in Response to Glucose Is Not Impaired in People with Type 2 Diabetes after Controlling for Glucose Disposal Rate. Diabetes.

[25] Kim H.W. (2021). Metabolomic Approaches to Investigate the Effect of Metformin: An Overview. Int. J. Mol. Sci..

[26] Scheen A.J. (1996). Clinical Pharmacokinetics of Metformin. Clin. Pharmacokinet..

[27] Ye Z., Wang S., Zhang C., Zhao Y. (2020). Coordinated Modulation of Energy Metabolism and Inflammation by Branched-Chain Amino Acids and Fatty Acids. Front. Endocrinol..

[28] Yoon M.-S. (2016). The Emerging Role of Branched-Chain Amino Acids in Insulin Resistance and Metabolism. Nutrients.

[29] Sjögren R.J.O., Rizo-Roca D., Chibalin A.V., Chorell E., Furrer R., Katayama S., Harada J., Karlsson H.K.R., Handschin C., Moritz T. (2021). Branched-Chain Amino Acid Metabolism Is Regulated by ERRα in Primary Human Myotubes and Is Further Impaired by Glucose Loading in Type 2 Diabetes. Diabetologia.

[30] Newgard C.B. (2012). Interplay between Lipids and Branched-Chain Amino Acids in Development of Insulin Resistance. Cell Metab..

[31] Lynch C.J., Adams S.H. (2014). Branched-Chain Amino Acids in Metabolic Signalling and Insulin Resistance. Nat. Rev. Endocrinol..

[32] Vanweert F., Schrauwen P., Phielix E. (2022). Role of Branched-Chain Amino Acid Metabolism in the Pathogenesis of Obesity and Type 2 Diabetes-Related Metabolic Disturbances BCAA Metabolism in Type 2 Diabetes. Nutr. Diabetes.

[33] Geidenstam N., Spégel P., Mulder H., Filipsson K., Ridderstråle M., Danielsson A.P.H. (2014). Metabolite Profile Deviations in an Oral Glucose Tolerance Test-a Comparison between Lean and Obese Individuals. Obesity.

[34] Wang Q., Jokelainen J., Auvinen J., Puukka K., Keinänen-Kiukaanniemi S., Järvelin M.R., Kettunen J., Mäkinen V.P., Ala-Korpela M. (2019). Insulin Resistance and Systemic Metabolic Changes in Oral Glucose Tolerance Test in 5340 Individuals: An Interventional Study. BMC Med..

[35] Sun Y., Gao H.Y., Fan Z.Y., He Y., Yan Y.X. (2020). Metabolomics Signatures in Type 2 Diabetes: A Systematic Review and Integrative Analysis. J. Clin. Endocrinol. Metab..

[36] Arneth B., Arneth R., Shams M. (2019). Metabolomics of Type 1 and Type 2 Diabetes. Int. J. Mol. Sci..

[37] Yang Q., Vijayakumar A., Kahn B.B. (2018). Metabolites as Regulators of Insulin Sensitivity and Metabolism. Nat. Rev. Mol. Cell Biol..

[38] Park J.-E., Lim H.R., Kim J.W., Shin K.-H. (2018). Metabolite Changes in Risk of Type 2 Diabetes Mellitus in Cohort Studies: A Systematic Review and Meta-Analysis. Diabetes Res. Clin. Pract..

[39] Liu X., Gao X., Zhang R., Liu Z., Shen N., Di Y., Fang T., Li H., Tian F. (2020). Discovery and Comparison of Serum Biomarkers for Diabetes Mellitus and Metabolic Syndrome Based on UPLC-Q-TOF/MS. Clin. Biochem..

[40] Palmer N.D., Stevens R.D., Antinozzi P.A., Anderson A., Bergman R.N., Wagenknecht L.E., Newgard C.B., Bowden D.W. (2015). Metabolomic Profile Associated with Insulin Resistance and Conversion to Diabetes in the Insulin Resistance Atherosclerosis Study. J. Clin. Endocrinol. Metab..

[41] Qiu G., Zheng Y., Wang H., Sun J., Ma H., Xiao Y., Li Y., Yuan Y., Yang H., Li X. (2016). Plasma Metabolomics Identified Novel Metabolites Associated with Risk of Type 2 Diabetes in Two Prospective Cohorts of Chinese Adults. Int. J. Epidemiol..

[42] Porcu E., Gilardi F., Darrous L., Yengo L., Bararpour N., Gasser M., Marques-Vidal P., Froguel P., Waeber G., Thomas A. (2021). Triangulating Evidence from Longitudinal and Mendelian Randomization Studies of Metabolomic Biomarkers for Type 2 Diabetes. Sci. Rep..

[43] Papandreou C., Hernández-Alonso P., Bulló M., Ruiz-Canela M., Li J., Guasch-Ferré M., Toledo E., Clish C., Corella D., Estruch R. (2020). High Plasma Glutamate and a Low Glutamine-to-Glutamate Ratio Are Associated with Increased Risk of Heart Failure but Not Atrial Fibrillation in the Prevención Con Dieta Mediterránea (PREDIMED) Study. J. Nutr..

[44] McCann M.R., de la Rosa M.V.G., Rosania G.R., Stringer K.A. (2021). L-Carnitine and Acylcarnitines: Mitochondrial Biomarkers for Precision Medicine. Metabolites.

[45] Makarova E., Makrecka-Kuka M., Vilks K., Volska K., Sevostjanovs E., Grinberga S., Zarkova-Malkova O., Dambrova M., Liepinsh E. (2019). Decreases in Circulating Concentrations of Long-Chain Acylcarnitines and Free Fatty Acids During the Glucose Tolerance Test Represent Tissue-Specific Insulin Sensitivity. Front. Endocrinol..

[46] Yoshimura K., Hirano S., Takata H., Funakoshi S., Ohmi S., Amano E., Nishi Y., Inoue M., Fukuda Y., Hayashi H. (2017). Plasma Mannose Level, a Putative Indicator of Glycogenolysis, and Glucose Tolerance in Japanese Individuals. J. Diabetes Investig..

[47] Taguchi T., Yamashita E., Mizutani T., Nakajima H., Yabuuchi M., Asano N., Miwa I. (2005). Hepatic Glycogen Breakdown Is Implicated in the Maintenance of Plasma Mannose Concentration. Am. J. Physiol.-Endocrinol. Metab..

[48] Gabbay K.H. (1975). Hyperglycemia, Polyol Metabolism, and Complications of Diabetes Mellitus. Annu. Rev. Med..

[49] Lanaspa M.A., Ishimoto T., Li N., Cicerchi C., Orlicky D.J., Ruzycki P., Rivard C., Inaba S., Roncal-Jimenez C.A., Bales E.S. (2013). Endogenous Fructose Production and Metabolism in the Liver Contributes to the Development of Metabolic Syndrome. Nat. Commun..

[50] Burns T.W., Terry B.E., Langley P.E., Robison G.A. (1979). Insulin Inhibition of Lipolysis of Human Adipocytes the Role of Cyclic Adenosine Monophosphate. Diabetes.

[51] Frape D.L., Williams N.R., Carpenter K.L.H., Freeman M.A., Palmer C.R., Fletcher R.J. (2000). Insulin Response and Changes in Composition of Non-Esterified Fatty Acids in Blood Plasma of Middle-Aged Men Following Isoenergetic Fatty and Carbohydrate Breakfasts. Br. J. Nutr..

[52] Nakamura H., Faludi G., Spitzer J.J. (1967). Changes of Individual Free Fatty Acids during Glucose Tolerance Test. Diabetes.

[53] Gilbert C.H., Kaye J., Galton D.J. (1974). The Effect of a Glucose Load on Plasma Fatty Acids and Lipolysis in Adipose Tissue of Obese Diabetic and Non-Diabetic Patients. Diabetologia.

[54] Rahman S.M.K., Uyama T., Hussain Z., Ueda N. (2021). Roles of Endocannabinoids and Endocannabinoid-Like Molecules in Energy Homeostasis and Metabolic Regulation: A Nutritional Perspective. Annu. Rev. Nutr..

[55] Chiazza F., Collino M. (2016). Peroxisome Proliferator-Activated Receptors (PPARs) in Glucose Control. Molecular Nutrition and Diabetes: A Volume in the Molecular Nutrition Series.

[56] Diamanti K., Cavalli M., Pereira M.J., Pan G., Castillejo-López C., Kumar C., Mundt F.O., Komorowski J., Deshmukh A.S., Mann M. (2022). Organ-specific metabolic pathways distinguish prediabetes, type 2 diabetes, and normal tissues. Cell Rep. Med..

[57] Boden G., Lebed B., Schatz M., Homko C., Lemieux S. (2001). Effects of Acute Changes of Plasma Free Fatty Acids on Intramyocellular Fat Content and Insulin Resistance in Healthy Subjects. Diabetes.

[58] Muoio D.M. (2014). Metabolic Inflexibility: When Mitochondrial Indecision Leads to Metabolic Gridlock. Cell.

[59] Lackey D.E., Lynch C.J., Olson K.C., Mostaedi R., Ali M., Smith W.H., Karpe F., Humphreys S., Bedinger D.H., Dunn T.N. (2013). Regulation of Adipose Branched-Chain Amino Acid Catabolism Enzyme Expression and Cross-Adipose Amino Acid Flux in Human Obesity. Am. J. Physiol. Endocrinol. Metab..

